# Open access journals are as likely to be referenced by the Orthopaedic literature, despite having a lower impact factor than subscription-based journals

**DOI:** 10.1051/sicotj/2021062

**Published:** 2021-12-21

**Authors:** Robert Cooke, Neil Jain

**Affiliations:** 1 Trauma and Orthopaedics Specialist Trainee – East Lancashire Hospitals Trust Haslingden Road Blackburn BB2 3HH UK; 2 Trauma and Orthopaedics Consultant, Department of Orthopaedics, Pennine Acute NHS Trust, Royal Oldham Hospital Rochdale Road Oldham OL1 2JH UK

**Keywords:** Publications, Open access, Orthopaedic Literature, Citations

## Abstract

*Background*: The internet has changed the way we access and publish Orthopaedic literature. Traditional subscription journals have been challenged by the open access method of publication which permits the author to make their article available to all readers for free, often at a cost to the author. This has also been adopted in part by traditional subscription journals forming hybrid journals. One of the criticisms of open access publications is that it provides the author with a “pay to publish” opportunity. We aimed to determine if access to the journals impacts their influence. *Methods*: We selected the top 40 Trauma and Orthopaedic Journals as ranked by the SCImago Rank. Each journal was reviewed and assessed for the journal quality, defined by reviewing the journal impact factor and SCImago rank; influence, defined by reviewing the top 10 articles provided by the journal for the number of citations; and cost of open access publication. *Results*: Of the top 40 journals, 10 were subscription, 10 were open access, and 20 were hybrid journals. Subscription journals had the highest mean impact factor, and SCImago rank with a significant difference in the impact factor (*p* = 0.001) and SCImago rank (*p* = 0.021) observed between subscription and open access journals. No significant difference was seen between citation numbers of articles published in subscription and open access journals (*p* = 0.168). There was a positive correlation between the cost of publishing in an open access journal and the impact factor (*r* = 0.404) but a negative correlation between cost and the number of citations (*r* = 0.319). *Conclusion*: Open access journals have significantly lower quality measures in comparison to subscription journals. Despite this, we found no difference between the number of citations, suggestive of there being no difference in the influence of these journals in spite of the observed difference in quality.

## Introduction

Orthopaedics is an ever-evolving specialism with practice regularly being improved due to developing evidence and research. Research is the cornerstone of this evolution, as the premise is to find a solution to a problem or identify a new idea that can further improve current practice.

There is an ongoing drive for doctors to undertake research in current practice, and there are many reasons why this is the case. Such reasons include aiming to improve patient outcomes with better equipment, techniques, or standards of care. Research can often progress the understanding of pathology and theories behind improved practices. For the individual, publishing research can improve their reputation and stand within their field of interest [1]. Research is also currently a requirement of the orthopaedic training programme in the United Kingdom [2].

Due to the various reasons for undertaking research, there may emerge a conflict between the individual’s requirement to publish research and the standard of research [3]. Therefore, the overall “quality” of a research project may be of a lower standard, having minimal or no impact on practice.

Many well-established journals have a strong reputation for publishing only high-quality research [4]. This is often reflected by the impact factor of the journal. The impact factor is an index score calculated based on the yearly mean number of citations for articles published by a journal over two years [5]. These have been published in ranking order for readers to understand the impact or importance of a publication from these journals.

Traditionally readers would be required to subscribe to a journal to read the published research. Alternatively, the reader would be able to access a copy of the journal from their institution’s library [6]. Subscription to these journals was often dependent upon the popularity of the journal and the clinical importance of the research published within.

With the development of the Internet, the medium in which journal articles are published and distributed has changed drastically. This has provided the reader with an easier search for their desired topic and gain immediate access to published articles. The Internet has also enabled the reader to access a broader number of articles published across the world.

In turn, this change to greater availability of articles online has created “open access” journals. Open access journals provide free download articles to any reader which the journal has published. Instead of the reader paying to read the article, the author of the publication may have paid the journal a fee to publish the article so that it is available to all. Subsequently, open access journals could be criticised for providing a “pay to publish” culture, which provides the author the benefits of publishing at a cost. This method could be criticised for publishing lower quality research in comparison to subscription journals due to higher acceptance rates [3, 7, 8].

Some subscription journals now allow the author to publish via either open access or subscription methods. This has created three different types of journals based on their publishing methods. Subscription journals are those which once published the articles are only available to those readers who have subscribed to the journal via individual or institutional access. Open access journals are those in which the reader and journal have made their article available to all readers free of charge. Hybrid journals adopt both methods of publication and provide the author of the article with the option to publish in either format.

This study aimed to determine if open access journals are now as influential as subscription-based journals.

## Method

We selected the top 40 Trauma and Orthopaedic Journals as ranked by the SCImago Journal and Country Rank as of January 2020. We excluded the journals which were based on sports medicine and basic science.

The SCImago Journal and Country Rank is an online portal that is publicly available to enable analysis and comparison between journals based on journal metrics [9].

For each journal, we collected data from reviewing freely available online material. Firstly, we reviewed the publication method of the journal from its “Author’s Instructions”. If it was identified as being a journal that provided open access publishing, then its cost was recorded. The Impact Factor, SCImago Rank, and H-index were recorded for each journal online. Each of the top 40 journals websites were reviewed for a list of their “Top 10” most popular, downloaded, or viewed articles. Each of these articles was reviewed for the article publication method and the number of citations recorded on Google Scholar. The number of downloads, views, or captures was also recorded from the article publication page on the journal’s website.

The data collected was then analysed and sub-categorised to identify if any statistical differences could be seen. Firstly, the journals were categories into their publication method of subscription, open access, or hybrid. These categories were then compared for journal quality, journal influence, and publication cost. We defined journal quality as the higher the Impact Factor, SCImago Rank, and H-index, the higher the quality of a journal. Journal influence is the number of citations a published article receives. The publication cost is the amount the author would have to pay in order for their article to be published.

## Results

Of the top 40 journals, 10 were subscription, 10 open access, and 20 hybrid journals. These are listed in Table 1 in order of SCImago Journal Rank. It was only possible to review articles for 30 of the 40 journals as 10 journals did not provide a list of their most read, downloaded, or viewed articles. In total, this provided 244 articles for review, with journals providing varying numbers of their most popular, downloaded, or viewed articles. Of the 244 articles available, 87 were published via open access and free to the reader.

**Table 1:** Journals reviewed by publication method listed by SCImago Journal Rank.

Subscription Journals	Open Access Journals	Hybrid Journals
Arthroscopy – Journal of Arthroscopic and Related Surgery	Acta Orthopaedica	Current Trauma Reports
Clinical Orthopaedics and Related Research	Bone and Joint Research	Bone and Joint Journal
International Orthopaedics	Journal of Orthopaedics and Traumatology	Journal of Arthroplasty
Journal of Orthopaedic Trauma	International Journal of Shoulder Surgery	Journal of Bone and Joint Surgery – Series A
Foot and Ankle International	International Journal of Spine Surgery	Journal of Shoulder and Elbow Surgery
European Spine Journal	World Journal of Orthopaedics	Knee Surgery, Sports Traumatology & Arthroscopy
The Journal of American Academy of Orthopaedic Surgeons	Advances in Orthopedics	Spine
Journal of Orthopaedic Research	Journal of Foot and Ankle Research	Orthopaedic Clinics of North America
Journal of Spinal Disorders and Techniques	Journal of Orthopaedic Surgery and Research	Archives of Orthopaedics and Trauma Surgery
Journal of Hand Surgery	CiOS – Clinics in Orthopedic Surgery	Journal of Orthopaedic Trauma
		Spine Journal
		Knee
		Journal of Paediatric Orthopaedics
		The Journal of Knee Surgery
		Orthopaedics and Traumatology: Surgery and Research
		Orthopedics
		Injury
		Clinical Spine Surgery
		HIP International
		European Journal of Orthopaedic Surgery and Traumatology

### Journal quality

The Subscription journals had the highest mean impact factor, SCImago rank, and H index. Open access journals had the lowest in all three of these categories (Table 2).

**Table 2:** Mean values of quality measures for subscription, open access and hybrid journals.

Journal type	Mean impact factor	Mean SCImago rank	Mean *H* index
Subscription	2.859	1.434	115.30
Open Access	1.717	1.040	36.50
Hybrid	2.327	1.403	95.95

T-Tests were performed between the different journal types for impact factor and SCImago rank to assess any statistical difference. A significant difference between the impact factor and SCImago rank was seen between subscription and open access journals (*p* = 0.01 and *p* = 0.021 respectively). No other statistical differences were seen between the journal types.

### Journal influence

Subscription journals had the highest number of citations and mean citations per article compared to Open Access and Hybrid Journals (Table 3). Despite the higher number of citations, it was not statistically significant.

**Table 3:** Collective data for the articles provided for each journal type.

Journal type	Journals with data	Total articles	Total citations	Mean citations per article	Mean articles per journal	Range of years of publication	Median year of article publication
Subscription	8	69	19,522	282.9	8.625	1993–2019	2015
Open access	5	30	8031	267.7	6	1969–2019	2012
Hybrid	17	145	7858	54	8.529	2000–2020	2018

The publication method of individual articles of either subscription or open access for all journal types was compared. This allowed a comparison between a greater number of subscription and open access articles, demonstrating a greater difference in mean citations per article (Table 4), but this was still not a statistical difference.

**Table 4:** Citations of different publication methods (*p* = 0.168).

Publication method	Total articles	Total citations	Mean citations per article
Subscription	157	29,152	185.7
Open access	87	9308	107.0

Subscription-only journals demonstrated a higher mean of citations per article than the subscription articles within a hybrid journal. Open access only journals had a higher mean citation per article than open access articles available within hybrid journals. These differences, however, were not statistically significant.

### Publication cost

Publishing within an open access journal requires a mean cost of publication of £1007 with a range of £0–£1790. Hybrid journals had a higher mean cost for open access publication at £2300 (range £1922–£2646). Spearman rank correlations between the cost of open access publishing and the journal quality measures were observed for Impact Factor (*r* = 0.404), SCImago Rank (*r* = 0.316), and *H* index (*r* = 0.534). Each of these demonstrates a strong positive correlation.

There was a negative correlation between the cost of open access publication and the mean number of citations for the journal (*r* = −0.319).

## Discussion

### Journal quality

We determined journal quality on the impact factor, SCImago journal rank, and *H* index, the higher the journal scored for these scoring systems, the higher the journal quality. These scoring systems enable the reader to compare “like-for-like” journals based on their journal metrics. The premise of these scoring systems is that they are based on the average number of citations per article within the journal over a period of time. The quality measures (impact factor, SCImago Journal Rank, and *H* index) were all significantly greater for the purely subscription journals than the purely open access journals. Additionally, there was a significantly greater impact factor observed for hybrid journals than purely open access journals. This suggests that journals publishing articles with some element of subscription (subscription only or hybrid) have an average higher quality of articles than those exclusively open access.

However, the hybrid journals had no statistical difference in quality measures to the subscription journals. Therefore, we can surmise that publishing open access articles alongside subscription articles in hybrid journals do not have a detrimental effect on these quality measures compared to subscription-only journals. Therefore, the mode of publication does not have an impact on the scoring of these quality measures.

The lower quality measures for the open access journals alongside a cost to the author are supportive of the theory of paying for publication. This theory has been surmised as the articles published in open access journals have lower average journal metrics, so therefore presumed to be of lower quality. Open access journals rely on publishing papers in order to make a financial profit. It could be hypothesised, therefore, that in order to increase their profit, they may have a higher acceptance rate of papers for publication, as the number of people who read the article does not impact the journal’s profit.

Open access is a newer form of publication method in comparison to the traditional subscription method. This, therefore, may provide the traditional subscription journals with greater perceived importance within the orthopaedic community. This could lead to readers citing these articles more frequently in their own work as they feel that these papers are of greater quality. Are the traditional journals article’s promoted better, so readers are more likely to see them than articles in lesser well-known journals.

An author’s desire to publish can be multifactorial. These include the fact that publications are currently one of the criteria required by Orthopaedic Specialist Trainees in the United Kingdom to enable them to receive their Certificate of Completion of Training (CCT) [2]. Furthermore, there is the need for every surgeon to maintain Continual Professional Development (CPD) and demonstrate this as part of their appraisal process [10]. Individually publications provide the authors with the “kudos” of having their work published [1] and can be used as reference when demonstrating an author as a key opinion leader [11].

### Journal influence

Journal influence was defined as the number of citations achieved for the top 10 articles published by the journal. When we reviewed the number of citations for open access and subscription journals, we found no significant difference between the two types of journals. The hybrid journals had the lowest mean number of citations of all three journal types. However, when the hybrid journal articles were separated into those which were published by subscription and open access and added to the respective groups, there remained no significant statistical difference between the publication methods.

Open access articles are more readily available to the reader as they are free to access. This greater ease in availability may mean they have as good a chance of impacting practice as articles in higher-ranked subscription journals. Our results show no statistical difference between the citations achieved for the top 10 articles of open access and subscription journals. Therefore, publishing via open access which is statistically lower-ranked journals, can provide as much influence as publishing in higher impact subscription journals [4]. However, this in turn could lead to the potential danger of lower quality articles having a greater effect than they should on clinical practice. This highlights the importance of the reader critically analysing individual papers to assess the validity of the conclusions.

The ability of readers to search for articles easily by keywords on databases such as PubMed and Google Scholar has promoted open access articles as the reader can immediately access these articles. In comparison to subscription publications which can be found as easily but may not be instantly accessible.

Our sample size from each journal to assess the influence of the journal is relatively small, but we feel it is reflective of the journal articles available. It would be interesting to see if this trend was matched in other specialities other than orthopaedics. Also, we should comment that a completely fair comparison between the number of downloads, views, and captures could not be performed as the metrics each journal provided were different.

### Publication cost

The cost of publishing for each open access journal was ascertained from reviewing the online author’s instructions to identify the fee. In most cases, there is a significant amount of money required, at the author’s expense, to publish an article as open access. We identified that there is a positive correlation between the impact factor and the price of publishing for open access articles. This highlights that in order to have work published in a higher-ranked journal, this comes at a greater financial cost to the author. This again may suggest that paying for open access may provide a pay to publish facility, as to have an article in higher-ranked journal is at greater cost to the author.

There is no data available within the public domain regarding acceptance rates for each journal, so it is therefore unclear if it is easier to be accepted for publication in open access in comparison to subscription journals in orthopaedics. This information would potentially be very valuable to the reader and the author. It would demonstrate the standard of acceptance threshold for each journal as well as provide the author with an understanding of their chances of successful submission. Such information could then be compared to the type of Journal, Subscription or Open Access, and compared to any publication fee to either support or reject the theory that it is easier to publish work in open access as is thought to potentially be the case [4, 7].

Our data identified that publishing by open access in a more expensive journal may provide your publication in a higher impact journal, but this does not necessarily provide more citations.

## Conclusions

An author publishing their work in an open access journal may be able to achieve as great an impact in the orthopaedic community as if they published in a higher-ranked subscription journal. The articles published in subscription and hybrid journals have a greater average number of citations and higher quality measures. However, the potential influence of the article is not dependent upon the type of journal it is published within.

Published acceptance rates of publications submitted to journals potentially could provide the reader with a greater understanding of the acceptance threshold for individual journals.

The high costs for the author associated with open access journals may be a barrier to their ability to publish significant findings, and with the importance attached to clinicians’ involvement in research, this could result in individual financial concerns.

In an era of evidence-based medicine driving improvements in clinical practice and patient outcomes, it may be argued that all publications should be available to readers so they can make the most informed decisions for their practice. The importance of a reader’s ability to critically analyse an article remains the most important factor in determining their own opinion in use within their practice.

## Conflict of interest

The authors declare that they have no relevant financial or non-financial interests to report.

## Funding

No funding support was obtained to assist with this manuscript.

## Ethical approval

No ethical approval was sought for this paper as there was no patient involvement, and all data were obtained from freely available information on the internet.

## Informed consent

Not applicable as no patients involved in the paper.

## Authors Contributions

Robert Cooke was involved in conceptualisation, methodology, data collection, writing, reviewing and editing. Neil Jain was involved in supervision, conceptualisation, methodology, writing, reviewing, and editing.
